# Multilevel diabetic foot revascularization in COVID 19 patient: Case report

**DOI:** 10.1016/j.ijscr.2021.106132

**Published:** 2021-06-24

**Authors:** E. Dinoto, F. Pecoraro, F. Ferlito, G. Tortomasi, D. Mirabella, G. Bajardi

**Affiliations:** a Vascular Surgery Unit, AOUP Policlinico ‘P. Giaccone’, Palermo, Italy; b University of Palermo, Department of Surgical, Oncological and Oral Sciences (Di.Chir.On.S.), Palermo, Italy

**Keywords:** Hybrid procedure, Peripheral arterial disease, COVID-19, Diabetic foot, Case report

## Abstract

**Introduction:**

Coronavirus 2019 (COVID-19) has been associated with endothelial dysfunction. This hypercoagulable state coming from the endothelial injury pones COVID-19 patients to a higher risk for thrombosis. COVID 19 diabetic patients are more exposed to peripheral vascular disease progression. Multilevel peripheral arterial disease is the main cause of critical limb ischemia. Vascular interventions are required to increase distal blood flow and reduce the risk of amputation.

**Presentation of case:**

We report a case of complex revascularization in a diabetic patient with aggressive right foot lesions evolution after COVID-19 infection. The patient presenting a Peripheral arterial ischemic involving the infrarenal aorta, iliac, femoral. The simultaneous intervention consisted of an endovascular aortic stent-graft placement and angioplasty of femoral artery.

**Discussion:**

Diabetes is a risk factor of severity and deaths in patients infected with pulmonary viruses. In our experience, COVID 19 virus can accelerate the ulcers generation and progression in diabetic patient. Hybrid interventions can be performed simultaneously or staged with benefit given by the complementary role of endovascular and surgical treatments. In the reported case, a complex simultaneous treatment in a patient presenting Multilevel peripheral arterial disease in association to COVID 19 infection was feasible in the same operation.

**Conclusion:**

Hybrid procedures are safe with high degree of efficacy in terms of revascularization, reduced morbidity and shorter intensive care. In our experience, the use of a hybrid procedure is technically feasible and allowed the treatment of complicated diabetic COVID-19 patient with a good outcome.

## Highlights

• COVID-19 has been associated with endothelial dysfunction and stasis.
• COVID 19 diabetic patient is more exposed to peripheral vascular disease progression.
• COVID 19 can accelerate the ulcers generation and progression in diabetic patient.
• Hybrid procedures can integrate endovascular and surgical benefits.

## 1 Introduction

Multilevel peripheral arterial disease (PAD) in diabetic patients, is one of the most frequent causes of amputation. After major lower-extremity amputation, the cumulative mortality rate at 1 year is high to 30.7% [1]. The actual severe acute respiratory syndrome coronavirus 2 (SARS-CoV-2) pandemic, has proven to have a significant role in the development of vascular events due to coagulative alteration related to excessive inflammation, platelet activation, endothelial dysfunction, and stasis [2].

Vascular interventions in PAD patients have the role to increase foot blood flow enhancing cutaneous oxygen pressure, promoting wound healing and reduce ischemic state in lower limbs [3,4]. Here we report a case COVID-19 positive diabetic patient with already known multilevel PAD, including abdominal aorta, iliac artery and femoral artery. In this case, we have chosen a hybrid procedure including remote simultaneous multiple endovascular procedures from a surgical exposed common femoral artery to address multiple vascular lesions.

This work has been written in accordance with the SCARE criteria [5].

## 2 Case report

A 56-years-old woman with history of hypertension, decompensated diabetes mellitus, was referred to our hospital with critical limb ischemia (CLI) presenting rest pain and ulcer on the left leg and negative COVID-19. On physical examination, her blood pressure was 160/90 mmHg with 130 b/m. Auscultation of the abdomen revealed a mesogastrium systolic bruit. Electrocardiogram showed signs of ST segment depression without thoracic pain. Coronary angiography was positive for severe coronary artery disease with no indication to treatment. Duplex ultrasound (DUS) revealed widespread irregularities of the aortic wall with monophasic wave on right common femoral artery and direct flow on the left femoral axis; diffuse disease of both superficial femoral arteries (SFA) with distal occlusion in the left side.

**Fig. 1:**
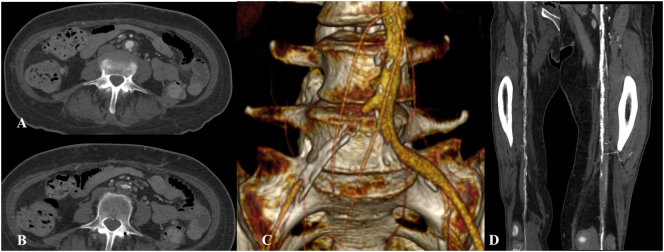
Preoperative CT-angiography showing significant atherosclerotic aortic disease (A and B), right common iliac artery (C) and femoral popliteal axes.

CT-scan showed atherosclerotic disease on infrarenal abdominal aorta with focal dissection, occlusion of right common iliac artery origin and short dissection of the left common iliac artery origin not hemodynamically significative (Fig. 1).

**Fig. 2:**
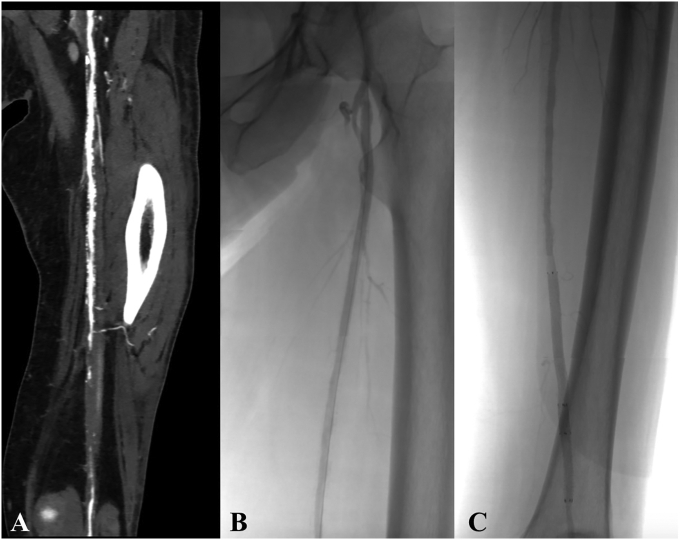
Preoperative CT-angiography showing atherosclerotic disease and occlusion of the left femoral artery (A); intraoperative angiography showing patency of superficial femoral artery after PTA with eluting ballon (B) and Stenting (C).

**Fig. 3:**
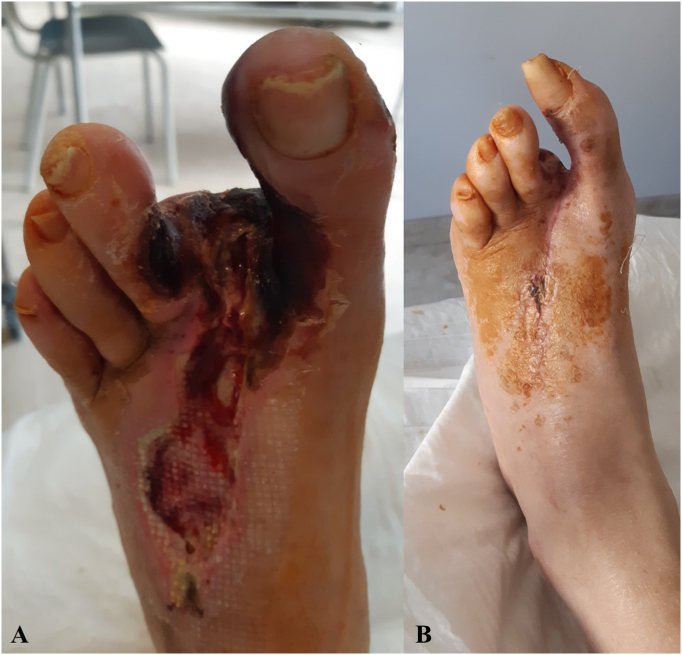
Left foot before revascolarizzation (A) and three week after (B).

The presence of ulcers in the left leg was the indication to address the left SFA throughout an antegrade percutaneous homolateral access with drug eluting balloon (DEB) angioplasty in the proximal SFA (6.0 × 60 mm Luminor®; iVascular, Vascular S.L.U., Barcelona, Spain) and distal stenting (5.0x80mm iVolution; iVascular) (Fig. 2). The final result was a complete lesion healing in the left foot at three weeks (Fig. 3).

After two months of the index procedure, the patient was readmitted due to gangrene of the right foot and asymptomatic COVID-19 infection. The control CT showed a similar vascular pattern on the right side with no significant difference with the previous CT. At this stage, the rapid and aggressive right foot lesions evolution was the indication to address vascular lesions in the right axis.

The intervention was carried under general anesthesia with surgical exposure of both common femoral arteries employed as remote accesses for multilevel endovascular intervention. The first step consisted of aorto-iliac repair (aortic dissection and right common iliac occlusion) using an AFX aortic endograft (Endoogix Inc., Irvine, CA, USA); stenting of the right common iliac artery (balloon expandable stent - Isthmus 10 × 39; CID SpA, Saluggia, Italy).

**Fig. 4:**
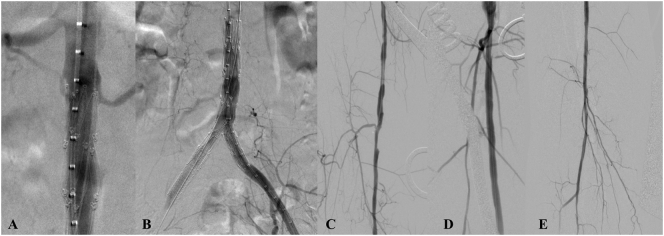
Completion intraoperative angiography showing the correct positioning and patency of the aortic stent-graft (A and B) with pre-PTA Angiograpfhy of SFA (C) and final outcome after PTA with eluting balloon (D and E).

**Fig. 5:**
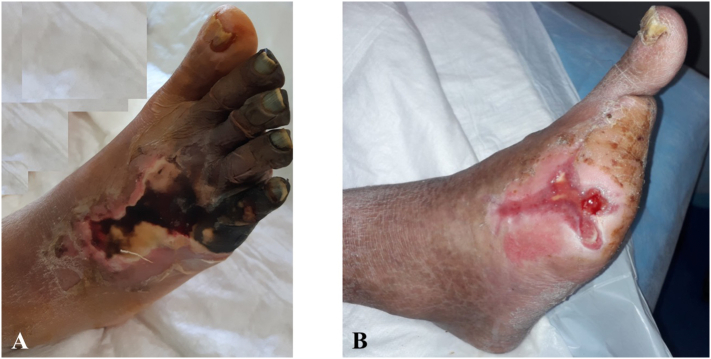
Right foot before revascolarizzation (A) and three week after (B).

During the same operation, the right SFA disease was addressed with a DEB (Luminor 5 × 200 iVascular) (Fig. 4). The multiple intervention determined the increase of the distal flow and the reappearance of dorsalis pedis pulse. After two days, the patient was asymptomatic for right foot pain and underwent to right foot minor amputation. The patient was discharged after a week with double antiplatelet therapy. CT angiography performed after two months confirmed the proper positioning of the aortic stent-graft with good patency of iliac-femoral axis and right foot ulcers healing (Fig. 5).

## 3 Discussion

The World Health Organization, on March 11, 2020, declared the SARS-CoV-2 an official pandemic. Thromboembolic complications, including pulmonary embolism (PE), peripheral venous and arterial thrombosis, and acute stroke (seen also in patients older than 50 years without risk factors) have been already reported [6].

Recent studies suggest that dysfunction of the endothelium during COVID-19 may exacerbate these deleterious events by inciting inflammatory and microvascular thrombotic processes. Endothelial dysfunction is also a common denominator in reported COVID-19 comorbidities. A case series of 5700 hospitalized COVID-19 patients reported that hypertension, obesity, and diabetes are the most common comorbidities, all of which involve underlying endothelial damage. Together, major clinical events in severe COVID-19 patients, laboratory evidence of endothelial dysfunction associated with poor prognosis, and reported comorbidities all support that COVID-19 targets endothelial cells [6]. Diabetes and uncontrolled glycemia were reported as significant predictors of severity and deaths in patients infected with different viruses, including the 2009 pandemic influenza A (H1N1), SARS-CoV and MERS-CoV. In the current SARS-CoV-2 pandemic, some studies did not find a clear association between diabetes and severe disease. However, other reports from China and Italy showed that older patients with chronic diseases, including diabetes, were at higher risk for severe COVID-19 and mortality. Diabetes is a chronic inflammatory condition characterized by multiple metabolic and vascular abnormalities that can affect our response to pathogens. Hyperglycemia and insulin resistance promote increased synthesis of glycosylation end products and pro-inflammatory cytokines, oxidative stress, in addition to stimulating the production of adhesion molecules that mediate tissue inflammation. This inflammatory process may compose the underlying mechanism that leads to a higher propensity to infections, with worse outcomes thereof in patients with diabetes [7].

However, is common experience that to increase the blood flow to the foot which in turn enhances cutaneous oxygen pressure promoting infection clearance and ulcer granulation [8,9]. Nowadays the use of endovascular therapies as primary treatment in patients with CLI and femoro-popliteal lesions is on the rise [10,11]. Infrarenal acute aortic syndromes (IAAS) can be managed with the medical, surgical, or endovascular approach. The endovascular approach showed better results with a reduced mortality and morbidity risk when compared to conventional open surgery. The main concerns of IAAS endovascular treatments are the lack of dedicated devices and the use of standard aortic stent-grafts outside the instruction for use (IFU). Different solutions including uncovered stents, covered stents, and stent-grafts have been proposed [12]. In literature, among the best commercially available device adapting to IAAS characteristic was the unibody stent-graft system (AFX endovascular AAA system; Endologix Inc.). The bottom-up construction of such a device allows its use in small aortic bifurcation diameter and short renal to aortic bifurcation distance [13], especially in women presenting higher incidence of such anatomia conditions [14].

Techniques such as subintimal angioplasty of the femoral-popliteal artery segment, retrograde angioplasty using trans-pedal access and arterial flossing with anterograde-retrograde intervention are relevant technical innovations that improve the percutaneous transluminal angioplasty success rate in the diabetic limb which, as open techniques, have the final outcome of increasing distal vascularization. Several studies have highlighted the importance of a deep revascularization in view of the fact that below the knee revascularization reduces the rate of non-healing, minor and major amputations [15].

In the reported case, the COVID-19 infection was determinant in accelerating the ulcers generation and progression. In our experience, the effectiveness of the revascularization is decisive factor that leads to the results to allow a reducing the levels of amputation.

## 4 Conclusions

In literature, COVID-19 is responsible not only for respiratory failure but also organ failure by multifactorial etiology with at the base an endothelial injury associated with dysfunction of microcirculation. COVID-19 in diabetic patient can accelerate the evolution of tissue damage.

Endovascular procedures have been used increasingly often for the treatment of multilevel vascular disease in the fragile vascular patient population. The results of the treatment seem to be with low morbidity. In our limited experience, the use of an endovascular approach for treatment of a complicated atherosclerotic peripheral decease in diabetic COVID-19 patient was safe and technically feasible with decrease of surgical and anesthetic risk.

## Funding

None.

## Ethical approval

None.

## Consent

Written informed consent was obtained from the patient for publication of this case report and accompanying images. A copy of the written consent is available for review by the Editor-in-Chief of this journal on request.

## Guarantor

Ettore Dinoto

## Provenance and peer review

Not commissioned, externally peer-reviewed.

## CRediT authorship contribution statement

Ettore Dinoto: study concept, design, data collection, data analysis, interpretation, writing the paper, final approval of the version to be submitted, guarantor.

Felice Pecoraro: study concept, design, data collection, data analysis, interpretation, writing the paper, final approval of the version to be submitted.

Francesca Ferlito: study concept, design, data collection, data analysis, interpretation, final approval of the version to be submitted.

Graziella Tortomasi: study concept, design, data collection, final approval of the version to be submitted.

Domenico Mirabella: study concept, design, data collection, final approval of the version to be submitted.

Guido Bajardi: study concept, design, data collection, data analysis, interpretation, final approval of the version to be submitted.

## Declaration of competing interest

The authors have no ethical conflicts to disclose.

## References

[1] Dinoto E, Pecoraro F, Mirabella D, Ferlito F, Farina A, Lo Biundo N, et al. A single-center experience on below-the-knee endovascular treatment in diabetic patients. Transl. Med. UniSa. 2020 Apr;21:21–3. PMC7039268 32123676

[2] Siddiqi HK, Libby P, Ridker PM. COVID-19 - a vascular disease. Trends Cardiovasc. Med. 2021 Jan;31(1):1–5. 10.1016/j.tcm.2020.10.005 PMC7556303 33068723

[3] Bracale U.M., Ammollo R.P., Hussein E.A., Hoballah J.J., Goeau-Brissonniere O., Taurino M. (2020). Managing peripheral artery disease in diabetic patients: a questionnaire survey from vascular centers of the Mediterranean Federation for the Advancing of vascular surgery (MeFAVS). Ann. Vasc. Surg..

[4] Dinoto E, Pecoraro F, Cutrupi A, Bracale UM, Panagrosso M, Bajardi G. Single staged hybrid approach for multilevel aortic-iliac-femoral-popliteal disease. Int. J. Surg. Case Rep. 2020;77S:S166–9. 10.1016/j.ijscr.2020.09.018 PMC7876840 33041255

[5] Agha R.A., Franchi T., Sohrabi C., Mathew G., for the SCARE Group (2020). The SCARE 2020 guideline: updating consensus Surgical CAse REport (SCARE) guidelines. Int. J. Surg..

[6] Revzin MV, Raza S, Warshawsky R, D'Agostino C, Srivastava NC, Bader AS, et al. Multisystem imaging manifestations of COVID-19, part 1: viral pathogenesis and pulmonary and vascular system complications. Radiogr. Rev. Publ. Radiol. Soc. N. Am. Inc. 2020 Oct;40(6):1574–99. 10.1148/rg.2020200149 PMC7534458 33001783

[7] Hussain A, Bhowmik B, do Vale Moreira NC. COVID-19 and diabetes: knowledge in progress. Diabetes Res. Clin. Pract. 2020 Apr;162:108142. 10.1016/j.diabres.2020.108142 PMC7144611 32278764

[8] COVIDSurg Collaborative, GlobalSurg Collaborative (2021 Mar 24). SARS-CoV-2 vaccination modelling for safe surgery to save lives: data from an international prospective cohort study. Br. J. Surg..

[9] Redlich U., Xiong Y.Y., Pech M., Tautenhahn J., Halloul Z., Lobmann R. (2011). Superiority of transcutaneous oxygen tension measurements in predicting limb salvage after below-the-knee angioplasty: a prospective trial in diabetic patients with critical limb ischemia. Cardiovasc. Intervent. Radiol..

[10] Bracale UM, Vitale G, Bajardi G, Narese D, Dinoto E, Giribono AM, et al. Use of the directional atherectomy for the treatment of femoro-popliteal lesions in patients with critical lower limb ischemia. Transl. Med. UniSa. 2016 Nov;15:42–7. PMC5120749 27896226

[11] Pecoraro F., Bajardi G., Dinoto E., Vitale G., Bellisi M., Bracale U.M. (2015). Endograft connector technique to treat popliteal artery aneurysm in a morbid obese patient. Vascular.

[12] Giribono AM, Ferrara D, Spalla F, Narese D, Bracale U, Pecoraro F, et al. Endovascular treatment of spontaneous isolated abdominal aortic dissection. Acta Radiol. Open. 2016 Dec;5(12):2058460116681042. 10.1177/2058460116681042 PMC5152934 27994881

[13] Pecoraro F., Dinoto E., Mirabella D., Ferlito F., Farina A., Pakeliani D. (2020). Endovascular treatment of spontaneous and isolated infrarenal acute aortic syndrome with unibody aortic stent-grafts. World J. Surg..

[14] Dinoto E, Pecoraro F, Ferlito F, Peluso A, Bajardi G. A rare case of infrarenal aortic coarctation in a young female. Int. J. Surg. Case Rep. 2020;77S:S152–6. 10.1016/j.ijscr.2020.07.083 PMC7876927 32888881

[15] Meloni M, Izzo V, Giurato L, Gandini R, Uccioli L. Below-the-ankle arterial disease severely impairs the outcomes of diabetic patients with ischemic foot ulcers. Diabetes Res. Clin. Pract. 2019 Jun;152:9–15. 10.1016/j.diabres.2019.04.031 31078668

